# Tracheostomy During Extracorporeal Membrane Oxygenation in Adult ICU Patients: A Systematic Review

**DOI:** 10.3390/jcm15093517

**Published:** 2026-05-04

**Authors:** Giuseppe Neri, Giuseppe Mazza, Jessica Ielapi, Helenia Mastrangelo, Federico Longhini, Vincenzo Bosco, Alessandro Russo, Francesca Serapide, Corrado Pelaia, Andrea Bruni, Eugenio Garofalo

**Affiliations:** 1 Department of Medical and Surgical Sciences, “Magna Graecia” University of Catanzaro, 88100 Catanzaro, Italy; giuseppeneri91@gmail.com (G.N.); giuseppe.mazza@unicz.it (G.M.); jessica.ielapi22@gmail.com (J.I.); flonghini@unicz.it (F.L.); vincenzo.bosco@unicz.it (V.B.); arusso@unicz.it (A.R.); f.serapide@unicz.it (F.S.); pelaia.corrado@unicz.it (C.P.); 2 ASP Catanzaro, 88100 Catanzaro, Italy; heleniamastrangelo@gmail.com; 3 Department of Pharmacy and Health and Nutrition Sciences, University of Calabria, 87036 Cosenza, Italy; andrea.bruni@unical.it

**Keywords:** extracorporeal membrane oxygenation, ECMO, tracheostomy, anticoagulation, bleeding, critical care, systematic review

## Abstract

**Background/Objectives:** Extracorporeal membrane oxygenation (ECMO) is increasingly used in adult critical care, but tracheostomy during ECMO remains controversial because of bleeding risk, anticoagulation exposure, and variability in patient selection. This systematic review evaluated the safety, timing, and clinical outcomes of tracheostomy in adult patients receiving ECMO. **Methods**: A systematic search of PubMed/MEDLINE was supplemented by additional searches in the Cochrane Library/CENTRAL and Scopus to identify studies evaluating tracheostomy in adult ECMO patients. The review was conducted according to PRISMA 2020 guidelines. After database searching and screening, 13 observational studies were included in the qualitative synthesis. **Results:** Across 13 studies encompassing 1918 patients, tracheostomy during ECMO was feasible and was not associated with procedure-related mortality. Bleeding was the main procedural complication, with reported rates varying according to study design, ECMO configuration, timing of tracheostomy, anticoagulation management, and bleeding definitions. Tracheostomy performed during active ECMO support was generally associated with a higher burden of bleeding or minor procedure-related complications than tracheostomy performed after decannulation. Tracheostomy was performed using percutaneous, surgical, open, or hybrid techniques, although comparative evidence between approaches remained limited. Early tracheostomy was associated with shorter ECMO duration, shorter mechanical ventilation, or improved clinical outcomes in selected cohorts, but timing definitions and outcome measures were heterogeneous. **Conclusions:** Tracheostomy during ECMO may support airway management, sedation reduction, and ventilatory progression, particularly in prolonged ECMO courses, but it carries a relevant bleeding and transfusion burden. Timing, anticoagulation management, patient selection, ECMO configuration, and procedural technique are likely to influence the risk-benefit balance. Prospective studies are needed to standardize definitions, compare techniques, and clarify optimal timing strategies.

## 1. Introduction

Extracorporeal membrane oxygenation (ECMO) has become an established rescue therapy for patients with severe acute respiratory or cardiopulmonary failure refractory to conventional management. Its use has markedly increased over the past decade, particularly following the H1N1 influenza pandemic and, more recently, during the COVID-19 era, where ECMO has demonstrated a survival benefit in selected patients with severe acute respiratory distress syndrome (ARDS) [1,2,3,4,5]. Despite advances in technology and patient selection, ECMO remains associated with substantial morbidity, including bleeding, thrombosis, and other treatment-related complications [6,7].

Prolonged invasive mechanical ventilation is common among ECMO-supported patients, often necessitating tracheostomy to facilitate ventilatory weaning, reduce sedation requirements, and improve patient comfort and airway management. In the general intensive care unit (ICU) population, tracheostomy has been associated with improved secretion clearance, reduced duration of mechanical ventilation, and facilitation of rehabilitation, although the optimal timing of the procedure remains debated [8,9,10,11]. However, its role in ECMO patients remains controversial because the usual advantages of tracheostomy must be balanced against the specific risks associated with extracorporeal support.

One of the main concerns in this setting is the requirement for systemic anticoagulation to maintain circuit patency, which significantly increases the risk of procedural bleeding. Hemorrhagic complications are among the most frequently reported adverse events during ECMO, and their clinical relevance extends beyond major hemorrhage to include transfusion burden, interruption of anticoagulation, and circuit-related consequences [6,7]. More broadly, the relevance of anticoagulation strategy in critically ill patients with severe respiratory failure is underscored by randomized data comparing bivalirudin and enoxaparin in intubated COVID-19 patients, highlighting the ongoing uncertainty regarding optimal anticoagulant management in high-risk populations [12].

Conversely, tracheostomy during ECMO may provide clinically relevant benefits. Observational ECMO-specific studies suggest that early tracheostomy may be associated with reduced duration of ECMO support and mechanical ventilation [13], likely through facilitation of sedation reduction and improved patient interaction. In selected patients, tracheostomy may also enable awake ECMO strategies, allowing spontaneous breathing and early mobilization during extracorporeal support [14].

ECMO patients are also exposed to additional complications, including infectious events, which may contribute to overall morbidity and clinical complexity [15].

Current evidence on tracheostomy in ECMO patients is limited by heterogeneity in patient populations, ECMO configurations, timing of tracheostomy, and anticoagulation management strategies. Moreover, available data suggest substantial variation in tracheostomy practice across centers, with no shared approach yet emerging for this high-risk population [16].

Therefore, a comprehensive synthesis of the available evidence is needed to better define the safety, optimal timing, and clinical impact of tracheostomy during ECMO support.

The aim of this systematic review is to evaluate the current literature on tracheostomy in adult patients receiving ECMO, focusing on procedural safety, complications—particularly bleeding—and relevant clinical outcomes, including duration of ECMO support, mechanical ventilation, and mortality.

## 2. Materials and Methods

### 2.1. Study Design and Selection

This systematic review was conducted in accordance with the Preferred Reporting Items for Systematic Reviews and Meta-Analyses (PRISMA) 2020 statement [17].

The review protocol was not registered.

The review question, eligibility criteria, and outcomes of interest were defined a priori, and study selection was structured according to the PICOS framework [18].

A systematic literature search was conducted in PubMed (MEDLINE) to identify studies evaluating tracheostomy in adult patients receiving extracorporeal membrane oxygenation (ECMO). To improve the comprehensiveness of the review, the search was subsequently extended to include the Cochrane Library/CENTRAL and Scopus using adapted search strategies. The search strategy combined Medical Subject Headings (MeSH) and free-text terms related to ECMO and tracheostomy, including extracorporeal membrane oxygenation/extracorporeal life support and tracheostomy/tracheotomy. The search was restricted to studies involving adult patients, articles published within the last 10 years, and those written in English. The final search was completed during the revision process. Overall, the PubMed/MEDLINE search yielded 164 records. Additional searches identified 15 records in the Cochrane Library/CENTRAL and 37 records in Scopus. The full PubMed/MEDLINE search strategy was as follows: (“extracorporeal membrane oxygenation” OR ECMO OR “extracorporeal life support” OR ECLS) AND (tracheostomy OR tracheotomy OR “percutaneous tracheostomy”). Adapted search strategies using the same core terms were applied in the Cochrane Library/CENTRAL and Scopus.

**Figure 1:**
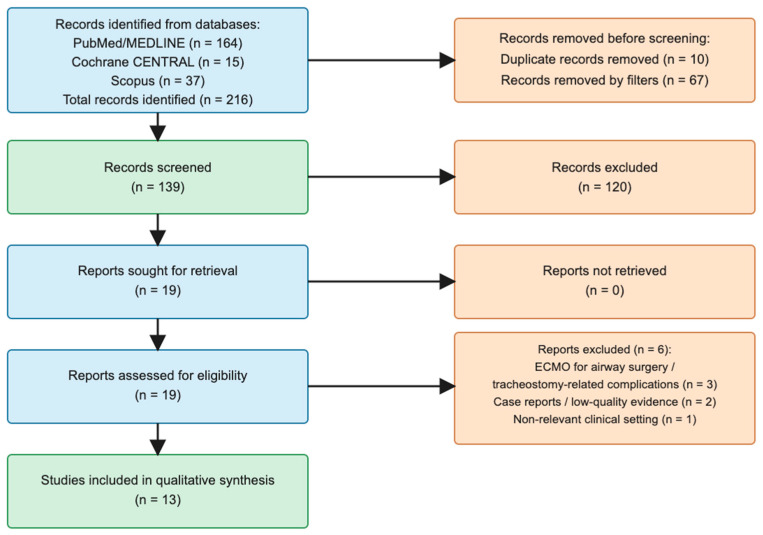
PRISMA 2020 flow diagram of study selection.

After application of the predefined filters, 97 PubMed/MEDLINE records remained for screening. Titles were reviewed for relevance to the review question, and records were excluded if they were unrelated to ECMO, did not include tracheostomy, involved pediatric populations, or represented review articles, editorials, or non-clinical studies. Two independent reviewers screened titles and abstracts for eligibility. Full-text articles were assessed independently, and any discrepancies were resolved by consensus. Data extraction was performed independently by two reviewers, and disagreements were resolved through discussion. Following title screening, 81 PubMed/MEDLINE records were excluded and 16 full-text articles were assessed for eligibility. Records identified through Cochrane Library/CENTRAL and Scopus were screened using the same eligibility criteria. Three additional eligible studies were identified through the extended database search and incorporated into the qualitative synthesis. During full-text evaluation, studies were excluded if ECMO was used exclusively for airway surgery or tracheostomy-related complications, if they were case reports, conference abstracts, pediatric studies, low-quality reports, or if they did not provide extractable clinical outcome data relevant to the review question. Ultimately, 13 studies were included in the qualitative synthesis. The study selection process is illustrated in Figure 1. The PRISMA 2020 checklist is provided as Supplementary Materials (Table S1).

### 2.2. Eligibility Criteria, Data Extraction and Outcomes

Eligibility criteria were defined according to the PICOS framework [18]. The population of interest consisted of adult patients admitted to the intensive care unit and supported with ECMO, either veno-venous (VV) or veno-arterial (VA), during the index hospitalization. The intervention was tracheostomy performed during ECMO support, irrespective of technique, including percutaneous dilatational, open, hybrid, and surgical tracheostomy. Comparators varied according to study design and included patients managed with versus without tracheostomy, patients undergoing early versus late tracheostomy, and patients receiving tracheostomy during ECMO versus after decannulation. Outcomes of interest included procedural safety, bleeding complications, transfusion requirements, duration of ECMO support, duration of mechanical ventilation, sedation requirements when available, length of stay, and mortality. Observational clinical studies with extractable quantitative data, including retrospective or prospective cohort studies and case series, were considered eligible, whereas case reports, review articles, editorials, experimental studies, and studies in which ECMO was used solely as support for airway surgery were excluded.

Data extraction was performed manually using a structured framework. For each included study, information was collected on study design, sample size, patient population, indication for ECMO, ECMO configuration, timing and technique of tracheostomy, anticoagulation management when reported, bleeding complications, transfusion requirements, duration of ECMO support, duration of mechanical ventilation, mortality, and additional clinical outcomes.

The primary outcome of the review was the safety of tracheostomy during ECMO, with particular emphasis on bleeding complications. Secondary outcomes included transfusion requirements, duration of ECMO support, duration of mechanical ventilation, sedation reduction, length of stay, and mortality. Comparisons were examined according to the data available within each study, with specific attention to differences between VV and VA ECMO populations, early versus late tracheostomy, tracheostomy performed during ECMO versus after decannulation, and tracheostomy versus no tracheostomy when a comparator group was available.

Given the observational design of the included studies, a formal risk of bias assessment was not performed; however, methodological quality and study limitations were considered during data interpretation.

### 2.3. Data Synthesis

Because the included studies differed substantially in design, patient selection, ECMO indication, tracheostomy timing, anticoagulation strategies, and outcome definitions, a meta-analysis was not performed. Instead, a qualitative synthesis was undertaken in accordance with general methodological recommendations for systematic reviews of heterogeneous observational studies [19]. Results were synthesized narratively and organized according to the main clinical domains of interest, including ECMO configuration, tracheostomy technique, anticoagulation management, safety and bleeding complications, transfusion requirements, timing of tracheostomy, and overall clinical outcomes.

The study selection process is illustrated in Figure 1. Due to the qualitative and heterogeneous nature of the included studies, a formal risk of bias assessment was not performed, as no standardized tool could be consistently applied across the different study designs. Artificial intelligence tools (Artificial intelligence tools (ChatGPT, GPT-5 model, OpenAI, San Francisco, CA, USA; https://chat.openai.com/, accessed on 7 April 2026) were used to assist in the preparation and revision of the PRISMA flow diagram and in language editing.) were used to assist in the drafting of the PRISMA flow diagram and in language editing.

## 3. Results

### 3.1. Study Characteristics

Thirteen studies met the inclusion criteria and were included in the qualitative synthesis. The main characteristics of the included studies are summarized in Table 1. All were observational, predominantly retrospective, and together accounted for 1918 patients. Sample size ranged from 24 patients in the smallest COVID-19 case series to 1168 patients in the largest international multicenter cohort [16,20]. Most studies were conducted in single-center settings and focused on adult ICU patients receiving ECMO for severe respiratory failure, particularly ARDS and COVID-19-related ARDS. However, the updated evidence base also included mixed VA/VV ECMO cohorts and predominantly VA-ECMO populations, thereby providing broader information on tracheostomy across different ECMO configurations [21,22,23,24]. Across the included studies, tracheostomy was most often evaluated in relation to procedural safety, bleeding risk, transfusion requirements, timing, and overall clinical outcomes.

### 3.2. VV Versus VA ECMO

Across the included studies, patients supported with VV-ECMO remained the most represented population, particularly in cohorts focused on ARDS or COVID-19-related respiratory failure [13,16,20,25,26,27,28,29]. These patients generally required prolonged invasive mechanical ventilation before tracheostomy and were managed in a clinical context dominated by refractory hypoxemia, prolonged sedation, secretion burden, and slow pulmonary recovery.

Within VV-ECMO cohorts, the proportion of patients undergoing tracheostomy varied widely, reflecting institutional practice and patient selection. In the comparative VV-ECMO cohorts, tracheostomy was performed in approximately 40% to 70% of eligible patients when reported, and the procedure was primarily undertaken to facilitate ventilatory weaning, reduce sedation burden, and optimize airway management rather than to address acute upper airway pathology [13,26,28,29]. Timing also varied, but in most studies tracheostomy was performed between day 5 and day 14 after ECMO initiation, consistent with prolonged ventilatory dependence in this population [13,16,27,29].

Compared with the original evidence base, the updated search identified additional mixed and predominantly VA-ECMO cohorts, providing broader information on tracheostomy across different ECMO configurations [21,22,23,24]. In the post-cardiac surgery cohort by Xin et al., tracheostomy timing was assessed in a predominantly cardiac ECMO population [21]. Similarly, Nukiwa et al. included a predominantly VA-ECMO cohort, whereas Jones et al. specifically reported outcomes in both VA- and VV-ECMO patients undergoing tracheostomy during ECMO or after ECMO explant [23,24]. In these settings, patients followed a different clinical course, often characterized by hemodynamic instability, vasopressor dependence, and the need to prioritize cardiac recovery over respiratory weaning.

Nevertheless, direct comparative evidence between VV- and VA-ECMO populations remains limited. Although Jones et al. provided stratified data according to ECMO modality, the available studies were not primarily designed to determine whether ECMO configuration independently modifies tracheostomy-related bleeding, transfusion burden, or survival [24]. Therefore, although tracheostomy during ECMO remains more commonly described in VV-ECMO populations, the updated evidence base suggests that the procedure is also feasible in selected VA-ECMO or mixed ECMO cohorts. Robust conclusions regarding differences in safety and clinical impact between VV- and VA-ECMO, however, cannot yet be drawn from the present evidence base.

### 3.3. Tracheostomy Technique

Across the included studies, percutaneous dilatational tracheostomy was the predominant procedural approach. Entire cohorts were managed with percutaneous techniques in several studies, including those by Xin et al., Kruit et al., and Dimopoulos et al. [21,25,27]. In the COVID-19 VV-ECMO series by Staibano et al., percutaneous tracheostomy accounted for 83.3% of procedures [20]. By contrast, explicit use of open or hybrid techniques was reported mainly in the studies by Jones et al. and Kelley et al., which included percutaneous, open, and hybrid tracheostomies in ECMO patients [24,28].

The available evidence does not allow a robust comparison between techniques, as most studies were either entirely percutaneous or did not stratify outcomes according to procedural approach. However, in the cohort by Kelley et al., post-procedural bleeding was observed across techniques and appeared numerically more frequent after percutaneous tracheostomy than after open or hybrid procedures, although the retrospective design and possible differences in patient selection preclude definitive conclusions [28]. Similarly, Jones et al. reported both percutaneous and open tracheostomy approaches in a mixed VA/VV ECMO cohort and found no difference in survival according to tracheostomy modality, although percutaneous tracheostomy was more frequently selected during active ECMO support [24]. Overall, the literature suggests that percutaneous tracheostomy is the most commonly adopted technique during ECMO, whereas comparative evidence between percutaneous and surgical approaches remains limited.

### 3.4. Anticoagulation Management

Anticoagulation management around the time of tracheostomy was variably reported across the included studies, and no uniform protocol emerged. Most patients remained on systemic unfractionated heparin during ECMO support, but peri-procedural strategies differed considerably between centers [16,25,27,28].

In the studies that described anticoagulation in more detail, tracheostomy was usually preceded by temporary reduction or interruption of heparin infusion. In Schmidt et al., heparin was typically stopped 4 h before the procedure and restarted approximately 2 h afterward if no major bleeding occurred [16]. In Kruit et al., anticoagulation management was similarly individualized, with variable heparin cessation before tracheostomy and early re-initiation after the procedure; however, no significant association was demonstrated between heparin interruption and the occurrence of post-procedural bleeding [25]. In the comparative transfusion study by Harris et al., the proportion of ECMO days without heparin and the number of heparin interruptions were not significantly different between tracheostomized and non-tracheostomized patients, suggesting that the increased blood product requirement observed in the tracheostomy group could not be explained by anticoagulation interruption alone [26].

Overall, the available data indicate that anticoagulation was almost always modified around tracheostomy, but the extent, timing, and monitoring of these modifications were inconsistently reported.

This heterogeneity makes it difficult to compare strategies across studies and likely contributes to the observed variability in bleeding outcomes. Recent large multicentre observational studies have further highlighted the complexity of anticoagulation and transfusion management during veno-venous ECMO, confirming the high incidence of bleeding complications and the lack of standardized strategies across centres [30,31]. In addition, the available studies primarily focused on unfractionated heparin, with limited data on alternative anticoagulation strategies (e.g., direct thrombin inhibitors such as bivalirudin or argatroban), precluding any meaningful comparison of their potential impact on bleeding risk.

A structured summary of bleeding definitions, bleeding outcomes, transfusion requirements, and anticoagulation management across the included studies is provided in Table 2.

### 3.5. Safety and Bleeding Complications

Tracheostomy performed during ECMO support was consistently reported as technically feasible and was not associated with procedure-related mortality in any of the included studies [16,25,27,28]. Additional mixed ECMO cohorts confirmed the procedural feasibility of tracheostomy during ECMO, with no procedure-related mortality reported [22,23,24]. Nevertheless, bleeding emerged as the most frequent procedural complication across the review.

The reported incidence of bleeding and bleeding-related complications varied substantially, depending on cohort characteristics, anticoagulation practices, ECMO configuration, timing of tracheostomy, and the definitions used to classify bleeding events [16,20,23,24,25,26,27,28,29]. In the study by Kruit et al., which specifically examined bleeding after percutaneous tracheostomy during VV-ECMO, the overall documented bleeding rate was 40%, with 32% minor bleeding and 8% significant bleeding [25]. In the single-center study by Dimopoulos et al., 10 of 65 patients (15%) developed at least one major complication, of whom 7 patients (11%) experienced major bleeding; in some of these cases, bleeding was accompanied by ECMO circuit dysfunction requiring oxygenator change [27]. In Kelley et al., 28 of 96 tracheostomized VV-ECMO patients developed post-procedural bleeding, including 9 major bleeding events and a larger number of minor airway or stoma-site bleeds [28]. In Nukiwa et al., bleeding requiring transfusion or intervention occurred in 13.3% of patients, without significant differences across tracheostomy timing quartiles [23]. Jones et al. reported major complications in 13 of 54 patients and found that minor complications were significantly more frequent when tracheostomy was performed during ECMO than after ECMO explant; however, these complications did not appear to reduce survival to hospital discharge [24]. Grewal et al. reported no major tracheostomy-related complications and no significant increase in packed red cell transfusion after tracheostomy [22]. In contrast, Staibano et al. reported a comparatively low perioperative bleeding burden in their COVID-19 VV-ECMO cohort, particularly in the context of withholding anticoagulation before and after the procedure [20]. Tamargo et al. also reported respiratory bleeding in their obese COVID-19 cohort but did not identify significant differences between early and late tracheostomy groups [29].

The clearest comparative evidence on bleeding risk was provided by the multicenter study by Schmidt et al., in which tracheostomy performed during active ECMO support was associated with a markedly higher incidence of local bleeding within 24 h than tracheostomy performed after ECMO decannulation (25% vs. 7%, *p* < 0.01) [16]. Across studies, bleeding events were predominantly local rather than catastrophic, and most were managed conservatively or with supportive measures. Taken together, the available evidence indicates that bleeding is common after tracheostomy in ECMO patients, but it is usually non-fatal and frequently manageable, with the highest risk observed when the procedure is performed under ongoing extracorporeal support.

### 3.6. Transfusion Requirements

Transfusion requirements were reported less consistently than bleeding rates but generally paralleled the burden of post-procedural hemorrhage. The most detailed comparison was provided by Harris et al., who showed that patients undergoing tracheostomy while on VV-ECMO required significantly higher daily red blood cell transfusion volumes than patients managed without tracheostomy (0.47 vs. 0.23 units/day; *p* = 0.02) and also had higher total blood product use (0.60 vs. 0.31 units/day; *p* = 0.01) [26]. In the same cohort, this increased transfusion exposure was not accompanied by significant differences in ECMO duration, weaning, or survival, suggesting that transfusion burden reflected procedural bleeding rather than overt differences in illness severity.

By contrast, Grewal et al. found no significant difference in packed red cell use after tracheostomy compared with the pre-tracheostomy period, suggesting that transfusion burden may vary substantially across centers and patient populations [22]. Kruit et al. similarly documented post-tracheostomy blood product administration in patients who bled after the procedure, including platelet transfusions in 6%, fresh frozen plasma in 2%, and packed red blood cells in 2% of cases [25]. In Kelley et al. and Dimopoulos et al., transfusion was incorporated into the definition of major bleeding, reinforcing the close relationship between hemorrhagic events and blood product exposure [27,28]. In Nukiwa et al. and Jones et al., transfusion was also embedded within the definition of bleeding or major complications, further highlighting the lack of standardized reporting across studies [23,24]. Although transfusion thresholds, reporting units, and peri-procedural anticoagulation policies were not standardized across studies, the overall pattern was consistent: when tracheostomy-related bleeding occurred, it frequently translated into additional transfusion requirements.

### 3.7. Timing of Tracheostomy

Timing of tracheostomy was one of the most clinically relevant and heterogeneously reported domains. The strongest direct early-versus-late comparison came from DiChiacchio et al., in which early tracheostomy, defined as placement within 7 days of ECMO initiation, was associated with a significantly shorter duration of ECMO support than late tracheostomy (12 vs. 21 days; *p* = 0.005) [13]. In the same study, time from ECMO initiation to discharge and to liberation from mechanical ventilation also tended to be shorter in the early tracheostomy group, although those differences did not consistently reach statistical significance [13]. Nukiwa et al. further evaluated tracheostomy timing in 98 ECMO patients and found a stepwise association between later tracheostomy and worse outcomes. Patients undergoing tracheostomy within 15 days had the lowest hospital mortality, whereas those undergoing tracheostomy after 26 days had the highest mortality; later tracheostomy timing was independently associated with increased hospital mortality [23]. Most tracheostomies in the included VV-ECMO cohorts were performed between day 5 and day 14 after ECMO initiation, suggesting that tracheostomy was generally considered once prolonged ventilatory dependence became established [13,16,27,29].

A different but equally important temporal comparison was provided by Schmidt et al., who examined tracheostomy performed during ECMO versus after ECMO decannulation [16]. In that study, tracheostomy during ECMO was associated with more frequent local bleeding, whereas tracheostomy performed after decannulation was associated with more rapid reductions in sedative and analgesic exposure and a higher level of consciousness in the early post-procedural period [16]. Jones et al. also compared tracheostomy during ECMO with tracheostomy after ECMO explant in a mixed VA/VV cohort, reporting more frequent minor complications during active ECMO support but no clear reduction in survival to hospital discharge [24]. By contrast, Tamargo et al. did not demonstrate significant differences in respiratory bleeding, ECMO duration, length of stay, or mortality between early and late tracheostomy in obese COVID-19 patients on VV-ECMO [29]. Overall, the literature suggests that timing is highly relevant, but it was defined inconsistently across studies, either by the number of days from ECMO initiation, the duration of mechanical ventilation before tracheostomy, or by the relation of the procedure to decannulation/explant, which limits direct comparison.

### 3.8. Clinical Outcomes

Clinical outcomes were reported variably across studies, but several recurring patterns emerged. Mortality in the COVID-19 VV-ECMO cohorts ranged from approximately 33% to 38%, as reported by Staibano et al. and Tamargo et al. [20,29]. In the comparative study by DiChiacchio et al., in-hospital mortality was numerically lower in the early tracheostomy group (9.1% vs. 24.1%) but did not reach statistical significance [13]. Nukiwa et al. reported a time-dependent association between later tracheostomy and higher mortality, with hospital mortality increasing from 19.2% in patients tracheostomized within 15 days to 50.0% in those tracheostomized after 26 days [23]. Likewise, Harris et al. and Kelley et al. did not identify consistent survival differences attributable to tracheostomy itself [26,28]. Jones et al. similarly found no clear survival disadvantage associated with tracheostomy during ECMO compared with no tracheostomy after correction for survival to explant, although patients undergoing tracheostomy had longer ventilator duration, likely reflecting selection of patients requiring prolonged support [24]. In the multicenter study by Schmidt et al., hospital survival was 67% in patients tracheostomized during ECMO and 87% in those tracheostomized after decannulation, although this comparison reflected differences in timing and clinical trajectory rather than an isolated effect of the procedure [16].

Beyond mortality, tracheostomy was repeatedly associated with effects on sedation and respiratory management. In Schmidt et al., cumulative sedative and analgesic use decreased more rapidly after tracheostomy when the procedure was performed after ECMO decannulation, and the delay to achieve an awake state was shorter than in patients tracheostomized during active ECMO support [16]. Grewal et al. observed a significant reduction in inotrope and vasopressor requirements after tracheostomy, together with a trend toward reduced analgesic use, suggesting potential benefits beyond airway management alone [22]. Swol et al. emphasized the role of tracheostomy in facilitating spontaneous breathing and awake ECMO strategies, thereby supporting lighter sedation and greater patient interaction with care [14]. DiChiacchio et al. further suggested that earlier tracheostomy may favor shorter ECMO runs and potentially earlier liberation from the ventilator [13]. Finally, Xin et al. reported shorter mechanical ventilation duration when percutaneous tracheotomy was performed during ECMO rather than after ECMO weaning in post-cardiac surgery patients, without a significant increase in tracheostomy-related complications [21]. Overall, while mortality findings were inconsistent, the available evidence more consistently suggests that tracheostomy may influence sedation exposure, airway management, and ventilatory course.

## 4. Discussion

### 4.1. Principal Findings

This systematic review shows that tracheostomy during ECMO is feasible and, in the available literature, not associated with procedure-related mortality, but it is performed in a setting characterized by a substantial burden of bleeding and transfusion requirements, marked variability in anticoagulation practice, and important differences in timing and patient selection [16,20,21,22,23,24,25,26,27,28,29]. The evidence also indicates that the current clinical experience is still mainly derived from VV-ECMO populations, although the updated synthesis includes additional mixed and predominantly VA-ECMO cohorts. Overall, the central issue is therefore not whether tracheostomy can be performed during ECMO, but rather in which patients, at what time, and under which peri-procedural conditions the risk-benefit balance is most favorable.

### 4.2. Clinical Interpretation

The main finding emerging from this review is that bleeding is the dominant procedural trade-off of tracheostomy during ECMO. This is consistent with the broader ECMO literature, where coagulopathy, platelet dysfunction, anticoagulant exposure, and circuit-related blood activation all contribute to a fragile hemostatic balance [4,6,7,32]. In this context, the higher bleeding burden observed in the included tracheostomy studies appears biologically plausible rather than unexpected. At the same time, most reported bleeding events were local and manageable rather than catastrophic, which suggests that tracheostomy should not be considered contraindicated per se during ECMO, but rather a high-risk procedure that requires protocolized peri-procedural management and center expertise [16,22,23,24,25,27,28].

The impact of bleeding, however, should not be judged only by mortality or overt major hemorrhage. Several of the included studies indicate that even non-fatal bleeding frequently translates into additional transfusion exposure, closer monitoring, and modifications in anticoagulation management [23,24,25,26,27,28]. In practical terms, this means that the burden of tracheostomy-related bleeding extends beyond the immediate procedure and may affect the overall ICU trajectory. This interpretation is reinforced by the growing ECMO literature on anticoagulant strategy, including recent data suggesting that anticoagulant choice may influence thrombotic complications and circuit-related events without necessarily eliminating bleeding risk [12,33].

The review also supports the concept that timing reflects a balance between competing clinical priorities. On one hand, earlier tracheostomy may facilitate airway management, improve tolerance of prolonged ventilation, reduce sedation burden, and possibly shorten ECMO duration or improve clinical outcomes in selected patients [13,23]. On the other hand, the multicenter data from Schmidt et al. indicate that tracheostomy performed during active ECMO is associated with substantially more bleeding than tracheostomy performed after decannulation [16]. Similarly, Jones et al. reported more frequent minor complications when tracheostomy was performed during ECMO rather than after ECMO explant, although this did not translate into reduced survival to hospital discharge [24]. These observations are not contradictory; rather, they describe the same clinical dilemma from different angles. Earlier tracheostomy may offer respiratory or organizational advantages, but these potential benefits must be weighed against the greater procedural vulnerability of the patient while still under full extracorporeal support.

Another important aspect is that the potential benefit of tracheostomy during ECMO is not limited to airway access alone. In selected patients, tracheostomy may fit into a broader strategy aimed at lighter sedation, improved interaction with care, spontaneous breathing, and functional recovery. This concept is indirectly supported by the included ECMO-specific studies and is consistent with the wider literature on early mobilization and spontaneous breathing during extracorporeal support [14,34,35]. In this context, Grewal et al. reported reduced inotrope and vasopressor requirements after tracheostomy, together with a trend toward reduced analgesic use, suggesting that the clinical effects of tracheostomy may extend beyond airway management alone [22]. Limited comparative data also suggest that procedural technique may contribute to outcome heterogeneity, as percutaneous tracheostomy predominated across the literature, whereas direct comparisons with open or hybrid approaches were rare and methodologically limited [20,21,22,23,24,25,26,27,28]. Accordingly, tracheostomy during ECMO should probably be viewed less as an isolated procedural decision and more as one component of a broader management pathway.

A further issue concerns generalizability. Most studies included in this review were conducted in VV-ECMO patients with severe respiratory failure, although the updated synthesis also includes mixed and predominantly VA-ECMO cohorts [21,23,24]. This distinction is clinically important because tracheostomy in VV-ECMO is usually considered in the context of prolonged ventilatory dependence, secretion management, and rehabilitation, whereas in VA-ECMO the immediate priorities are often hemodynamic stabilization and myocardial recovery. The broader adult ECMO literature also supports the view that respiratory and cardiac ECMO populations should not be interpreted as interchangeable when management decisions are considered [36]. Therefore, the conclusions of the present review should be applied most directly to respiratory ECMO populations, while recognizing that selected mixed and VA-ECMO cohorts provide additional but still limited evidence across other ECMO configurations.

### 4.3. Implications for Practice and Future Research

From a practical standpoint, these findings support a selective and individualized approach to tracheostomy during ECMO rather than a routine or reflexive strategy. In carefully selected patients—particularly those on prolonged VV-ECMO with persistent ventilatory dependence—tracheostomy may offer clinically meaningful advantages in airway management, sedation reduction, and progression toward spontaneous breathing. At the same time, the procedure should be embedded in a structured pathway that addresses anticoagulation interruption, bleeding surveillance, blood product availability, and multidisciplinary procedural planning.

The review also suggests that center-level organization probably matters as much as technical execution. High-volume ECMO centers may achieve acceptable safety profiles not only because of procedural expertise, but also because anticoagulation management, airway planning, and post-procedural monitoring are integrated within a coordinated clinical workflow. This likely explains part of the heterogeneity observed across the included studies.

Future research should address several unresolved issues. First, the field needs more consistent definitions of minor and major bleeding, as well as standardized reporting of transfusion thresholds and peri-procedural anticoagulation protocols. Second, prospective studies are needed to clarify whether specific timing strategies can improve respiratory outcomes without increasing procedural harm. Third, although the updated evidence base includes additional mixed and predominantly VA-ECMO cohorts, VA-ECMO populations remain less consistently studied than VV-ECMO populations and should be examined separately rather than extrapolated from respiratory ECMO data. Finally, future studies should move beyond mortality alone and incorporate outcomes that may be especially relevant in ECMO patients, including sedation burden, time to spontaneous breathing, mobilization, functional recovery, and the potential impact of different tracheostomy techniques.

### 4.4. Limitations

The findings of this review should be interpreted in light of several limitations. All included studies were observational and most were retrospective, making the available evidence vulnerable to selection bias, center effect, and confounding by indication. In addition, the included populations were heterogeneous, encompassing non-COVID ARDS, COVID-19-related respiratory failure, post-cardiac surgery patients, and mixed VA/VV ECMO cohorts, with important differences in clinical trajectory and indication for tracheostomy. Definitions of tracheostomy timing, bleeding complications, and transfusion thresholds also varied across studies, limiting direct comparison. Furthermore, although the updated synthesis includes additional mixed and predominantly VA-ECMO cohorts, most of the available evidence remains derived from VV-ECMO populations. Because of this heterogeneity in study design, patient selection, and outcome reporting, a meta-analysis was not feasible and the findings of this review should therefore be interpreted as qualitative rather than pooled quantitative evidence.

## 5. Conclusions

In adult ICU patients receiving ECMO, tracheostomy appears to be a feasible procedure that may support airway management, sedation reduction, and ventilatory progression, particularly in prolonged ECMO courses. However, it is consistently associated with a relevant burden of bleeding and transfusion requirements, especially when performed during active ECMO support. The currently available evidence suggests that timing, anticoagulation management, patient selection, ECMO configuration, and procedural technique are central determinants of the risk–benefit balance of the procedure. Given the predominance of observational data and the heterogeneity of ECMO populations, tracheostomy during ECMO should currently be considered a selective, individualized intervention best integrated within experienced multidisciplinary pathways. Further prospective studies are needed to clarify optimal timing, standardize anticoagulation strategies, better compare tracheostomy techniques, and define clinically meaningful outcomes beyond mortality alone.

## Figures and Tables

**Table 1:** Main characteristics of the included studies.

Study	Design/Population	ECMO Type	Main Comparison or Focus
DiChiacchio et al. [13]	Retrospective cohort, 50 ARDS patients	VV	Early vs. late tracheostomy
Swol et al. [14]	Retrospective cohort, 95 ECLS/ECMO surgical ICU patients	Mixed	Tracheostomy as bridge to spontaneous breathing/awake ECMO
Schmidt et al. [16]	International multicenter retrospective cohort, 1168 severe ARDS patients	Predominantly VV	Tracheostomy during ECMO vs. after decannulation
Staibano et al. [20]	Retrospective case series, 24 COVID-19 patients	VV	Descriptive cohort
Xin et al. [21]	Retrospective post-cardiac surgery cohort, 62 patients	Predominantly VA/mixed cardiac ECMO	Tracheostomy during ECMO vs. after ECMO weaning
Grewal et al. [22]	Single-center observational study31 tracheostomized ECMO patients from a cohort of 140 ECMO patients	Mixed	Safety and clinical effects of tracheostomy during ECMO
Nukiwa et al. [23]	Single-center retrospective observational study, 98 ECMO patients undergoing tracheostomy	Predominantly VA/mixed	Tracheostomy timing and clinical outcomes
Jones et al. [24]	Retrospective single-center cohort, 54 tracheostomized ECMO patients from 521 VA/VV ECMO patients	Mixed	Tracheostomy during ECMO vs. after ECMO explant; complications and clinical outcomes
Kruit et al. [25]	Retrospective cohort, 50 patients	VV	Bleeding after percutaneous tracheostomy
Harris et al. [26]	Retrospective comparative cohort, 63 patients	VV	Tracheostomy vs. no tracheostomy
Dimopoulos et al. [27]	Retrospective cohort, 65 patients	VV	Safety of percutaneous tracheostomy during VV-ECMO
Kelley et al. [28]	Retrospective cohort, 96 patients	VV	Safety and bleeding of tracheostomy during VV-ECMO
Tamargo et al. [29]	Retrospective cohort, 62 obese COVID-19 patients	VV	Early vs. late tracheostomy

Abbreviations: VV, veno-venous; VA, veno-arterial; ECMO, extracorporeal membrane oxygenation.

**Table 2:** Summary of bleeding and transfusion outcomes in studies evaluating tracheostomy during ECMO.

Study	ECMO Type	Bleeding Definition	Bleeding Outcomes	Transfusion Outcomes	Anticoagulation Management
DiChiacchio et al. [13]	VV	Not clearly defined	No major increase in bleeding reported	Not clearly reported	Not clearly reported
Swol et al. [14]	Mixed	Not reported	Not reported	Not reported	Not reported
Schmidt et al. [16]	Predominantly VV	Procedure-related bleeding	No significant increase in major bleeding events	Transfusions required in selected cases	Heparin stopped before procedure and restarted after
Staibano et al. [20]	VV	Not clearly defined	Limited bleeding complications reported	Not clearly reported	Not clearly reported
Xin et al. [21]	Predominantly VA/mixed	Procedure-related bleeding	Low incidence of bleeding complications	Not clearly reported	Not clearly reported
Grewal et al. [22]	Mixed Not clearly defined	Major tracheostomy-related complications reported	No major tracheostomy-related complications reported	No significant difference in packed red cell transfusion after tracheostomy	Not clearly reported
Nukiwa et al. [23]	Predominantly VA/mixed	Bleeding requiring blood transfusion or intervention Bleeding occurred in 13.3% of patients	no significant differences across timing quartiles	Included in bleeding definition	Not clearly reported
Jones et al. [24]	Mixed VA/VV	Minor and major tracheostomy complications	Major complications included transfusion-requiring events	Transfusion included in major complication definition	Not clearly reported
Kruit et al. [25]	VV	Major bleeding (not uniformly defined)	No significant increase in bleeding events	Not clearly reported	Anticoagulation managed individually
Harris et al. [26]	VV	Not clearly defined	Increased bleeding-related resource utilization in the tracheostomy group	Higher transfusion requirements in tracheostomy group	Not associated with anticoagulation interruption
Dimopoulos et al. [27]	VV	Procedure-related bleeding	Low incidence of major bleeding	Not clearly reported	Not clearly reported
Kelley et al. [28]	VV	Not clearly defined	No major increase in bleeding complications	Not clearly reported	Not clearly reported
Tamargo et al. [29]	VV	Not clearly defined	No significant increase in bleeding reported	Not clearly reported	Not clearly reported

## Data Availability

The data supporting the findings of this study are available within the article and its Supplementary Materials. Further inquiries can be directed to the corresponding author.

## Supplementary Materials

The following supporting information can be downloaded at https://www.mdpi.com/article/10.3390/jcm15093517/s1. Table S1: PRISMA 2020 checklist. Reference [17] is cited in Supplementary Materilas.

## Abbreviations

ARDS	Acute respiratory distress
COVID-19	Coronavirus disease 2019
ECMO	Extracorporeal membrane oxygenation
ECLS	Extracorporeal life support
ELSO	Extracorporeal Life Support Organization
ICU	Intensive care unit
MeSH	Medical Subject Headings
PDT	Percutaneous dilatational tracheostomy
PICOS	Population, Intervention, Comparison, Outcomes, Study design
PRISMA	Preferred Reporting Items for Systematic Reviews and Meta-Analyses
VA	Veno-arterial
VV	Veno-venous
VAP	Ventilator-associated pneumonia

## References

[1] Combes A., Hajage D., Capellier G., Demoule A., Lavoué S., Guervilly C., Da Silva D., Zafrani L., Tirot P., Veber B. (2018). Extracorporeal Membrane Oxygenation for Severe Acute Respiratory Distress Syndrome. N. Engl. J. Med..

[2] Peek G.J., Mugford M., Tiruvoipati R., Wilson A., Allen E., Thalanany M.M., Hibbert C.L., Truesdale A., Clemens F., Cooper N. (2009). Efficacy and economic assessment of conventional ventilatory support versus extracorporeal membrane oxygenation for severe adult respiratory failure (CESAR): A multicentre randomised controlled trial. Lancet.

[3] Barbaro R.P., MacLaren G., Boonstra P.S., Iwashyna T.J., Slutsky A.S., Fan E., Bartlett R.H., Tonna J.E., Hyslop R., Fanning J.J. (2020). Extracorporeal membrane oxygenation support in COVID-19: An international cohort study of the Extracorporeal Life Support Organization registry. Lancet.

[4] Tonna J.E., Abrams D., Brodie D., Greenwood J.C., Rubio Mateo-Sidron J.A., Usman A., Fan E. (2021). Management of Adult Patients Supported with Venovenous Extracorporeal Membrane Oxygenation (VV ECMO): Guideline from the Extracorporeal Life Support Organization (ELSO). ASAIO J..

[5] Munshi L., Walkey A., Goligher E., Pham T., Uleryk E.M., Fan E. (2019). Venovenous extracorporeal membrane oxygenation for acute respiratory distress syndrome: A systematic review and meta-analysis. Lancet Respir. Med..

[6] Bruni A., Battaglia C., Bosco V., Pelaia C., Neri G., Biamonte E., Manti F., Mollace A., Boscolo A., Morelli M. (2024). Complications during Veno-Venous Extracorporeal Membrane Oxygenation in COVID-19 and Non-COVID-19 Patients with Acute Respiratory Distress Syndrome. J. Clin. Med..

[7] Murphy D.A., Hockings L.E., Andrews R.K., Aubron C., Gardiner E.E., Pellegrino V.A., Davis A.K. (2015). Extracorporeal membrane oxygenation-hemostatic complications. Transfus. Med. Rev..

[8] Griffiths J., Barber V.S., Morgan L., Young J.D. (2005). Systematic review and meta-analysis of studies of the timing of tracheostomy in adult patients undergoing artificial ventilation. BMJ.

[9] McGrath B.A., Brenner M.J., Warrillow S.J., Pandian V., Arora A., Cameron T.S., Añon J.M., Hernández Martínez G., Truog R.D., Block S.D. (2020). Tracheostomy in the COVID-19 era: Global and multidisciplinary guidance. Lancet Respir. Med..

[10] Young D., Harrison D.A., Cuthbertson B.H., Rowan K., TracMan Collaborators (2013). Effect of early vs. late tracheostomy placement on survival in patients receiving mechanical ventilation: The TracMan randomized trial. JAMA.

[11] Hosokawa K., Nishimura M., Egi M., Vincent J.L. (2015). Timing of tracheotomy in ICU patients: A systematic review of randomized controlled trials. Crit. Care.

[12] Garofalo E., Cammarota G., Neri G., Macheda S., Biamonte E., Pasqua P., Guzzo M.L., Longhini F., Bruni A., BivaCOVID authors (2022). Bivalirudin vs. Enoxaparin in Intubated COVID-19 Patients: A Pilot Multicenter Randomized Controlled Trial. J. Clin. Med..

[13] DiChiacchio L., Boulos F.M., Brigante F., Raithel M., Shah A., Pasrija C., Mackowick K., Menaker J., Mazzeffi M., Herr D. (2020). Early tracheostomy after initiation of venovenous extracorporeal membrane oxygenation is associated with decreased duration of extracorporeal membrane oxygenation support. Perfusion.

[14] Swol J., Strauch J.T., Schildhauer T.A. (2017). Tracheostomy as a bridge to spontaneous breathing and awake-ECMO in non-transplant surgical patients. Eur. J. Heart Fail..

[15] Serapide F., Serraino R., Feola A., Morrone H.L., Olivadese V., Neri G., Biamonte E., Bruni A., Garofalo E., Longhini F. (2026). Invasive Fungal Infections During Extracorporeal Membrane Oxygenation: A Case Series from Intensive Care Unit and Literature Review. Diagnostics.

[16] Schmidt M., Fisser C., Martucci G., Abrams D., Frapard T., Popugaev K., Arcadipane A., Bromberger B., Lino G., Serra A. (2021). Tracheostomy management in patients with severe acute respiratory distress syndrome receiving extracorporeal membrane oxygenation: An International Multicenter Retrospective Study. Crit. Care.

[17] Page M.J., Moher D., Bossuyt P.M., Boutron I., Hoffmann T.C., Mulrow C.D., Shamseer L., Tetzlaff J.M., Akl E.A., Brennan S.E. (2021). PRISMA 2020 explanation and elaboration: Updated guidance and exemplars for reporting systematic reviews. BMJ.

[18] Richardson W.S., Wilson M.C., Nishikawa J., Hayward R.S. (1995). The well-built clinical question: A key to evidence-based decisions. ACP J. Club.

[19] Cumpston M., Li T., Page M.J., Chandler J., Welch V.A., Higgins J.P., Thomas J. (2019). Updated guidance for trusted systematic reviews: A new edition of the Cochrane Handbook for Systematic Reviews of Interventions. Cochrane Database Syst. Rev..

[20] Staibano P., Khattak S., Amin F., Engels P.T., Sommer D.D. (2023). Tracheostomy in Critically Ill COVID-19 Patients on Extracorporeal Membrane Oxygenation: A Single-Center Experience. Ann. Otol. Rhinol. Laryngol..

[21] Xin M., Wang L., Li C., Hou D., Wang H., Wang J., Jia M., Hou X. (2023). Percutaneous dilatation tracheotomy in patients on extracorporeal membrane oxygenation after cardiac surgery. Perfusion.

[22] Grewal J., Sutt A.L., Cornmell G., Shekar K., Fraser J.F. (2020). Safety and Putative Benefits of Tracheostomy Tube Placement in Patients on Extracorporeal Membrane Oxygenation: A Single-Center Experience. J. Intensive Care Med..

[23] Nukiwa R., Uchiyama A., Tanaka A., Kitamura T., Sakaguchi R., Shimomura Y., Ishigaki S., Enokidani Y., Yamashita T., Koyama Y. (2022). Timing of tracheostomy and patient outcomes in critically ill patients requiring extracorporeal membrane oxygenation: A single-center retrospective observational study. J. Intensive Care.

[24] Jones A., Olverson G., Hwang J., Bhagat R., McGann K., Bradburn K., Miller M., Louis C. (2022). The effect of tracheostomy on extracorporeal membrane oxygenation outcomes. J. Card. Surg..

[25] Kruit N., Valchanov K., Blaudszun G., Fowles J.A., Vuylsteke A. (2018). Bleeding Complications Associated With Percutaneous Tracheostomy Insertion in Patients Supported With Venovenous Extracorporeal Membrane Oxygen Support: A 10-Year Institutional Experience. J. Cardiothorac. Vasc. Anesth..

[26] Harris D.D., Shafii A.E., Baz M., Tribble T.A., Ferraris V.A. (2019). Increased blood transfusion and its impact in patients having tracheostomy while on extracorporeal membrane oxygenation. Perfusion.

[27] Dimopoulos S., Joyce H., Camporota L., Glover G., Ioannou N., Langrish C.J., Retter A., Meadows C.I.S., Barrett N.A., Tricklebank S. (2019). Safety of Percutaneous Dilatational Tracheostomy During Veno-Venous Extracorporeal Membrane Oxygenation Support in Adults With Severe Respiratory Failure. Crit. Care Med..

[28] Kelley K.M., Galvagno S.M., Wallis M., Mazzeffi M.A., Deatrick K., Betzold R., Scalea T., Menaker J. (2021). Tracheostomy in Patients on Venovenous Extracorporeal Membrane Oxygenation: Is It Safe?. Am. Surg..

[29] Tamargo I.A., Creel-Bulos C., Callahan M.C., Miller C., Dave S.B., Parrilla G.A., Chan J.L., Daneshmand M.A., Javidfar J. (2023). Early Tracheostomy May Be Performed Safely in Obese COVID-19 Patients Supported on VV-ECMO. Innovations.

[30] Martucci G., Giani M., Schmidt M., Tanaka K., Tabatabai A., Tuzzolino F., Agerstrand C., Riera J., Ramanan R., Grasselli G. (2024). Anticoagulation and bleeding during veno-venous extracorporeal membrane oxygenation: Insights from the PROTECMO study. Am. J. Respir. Crit. Care Med..

[31] Martucci G., Schmidt M., Agerstrand C., Tabatabai A., Tuzzolino F., Giani M., Ramanan R., Grasselli G., Schellongowski P., Riera J. (2023). Transfusion practice in patients receiving veno-venous extracorporeal membrane oxygenation (PROTECMO): A prospective, multicentre, observational study. Lancet Respir. Med..

[32] McMichael A.B.V., Ryerson L.M., Ratano D., Fan E., Faraoni D., Annich G.M. (2022). 2021 ELSO Adult and Pediatric Anticoagulation Guidelines. ASAIO J..

[33] Wieruszewski P.M., Macielak S.A., Nei S.D., Moman R.N., Seelhammer T.G., Nabzdyk C.G.S., Gerberi D.J., Mara K.C., Hooten W.M., Wittwer E.D. (2023). Heparin Versus Bivalirudin for Anticoagulation in Adult Extracorporeal Membrane Oxygenation: A Systematic Review and Meta-Analysis. ASAIO J..

[34] Abrams D., Javidfar J., Farrand E., Mongero L.B., Agerstrand C.L., Ryan P., Zemmel D., Galuskin K., Morrone T.M., Boerem P. (2014). Early mobilization of patients receiving extracorporeal membrane oxygenation: A retrospective cohort study. Crit. Care.

[35] Crotti S., Bottino N., Spinelli E. (2018). Spontaneous breathing during veno-venous extracorporeal membrane oxygenation. J. Thorac. Dis..

[36] Abrams D., Combes A., Brodie D. (2014). Extracorporeal membrane oxygenation in cardiopulmonary disease in adults. J. Am. Coll. Cardiol..

